# Profiling donepezil template into multipotent hybrids with antioxidant properties

**DOI:** 10.1080/14756366.2018.1443326

**Published:** 2018-03-13

**Authors:** Eva Mezeiova, Katarina Spilovska, Eugenie Nepovimova, Lukas Gorecki, Ondrej Soukup, Rafael Dolezal, David Malinak, Jana Janockova, Daniel Jun, Kamil Kuca, Jan Korabecny

**Affiliations:** a Biomedical Research Centre, University Hospital Hradec Kralove, Hradec Kralove, Czech Republic;; b National Institute of Mental Health, Klecany, Czech Republic;; c Department of Toxicology and Military Pharmacy, Faculty of Military Health Sciences, Hradec Kralove, Czech Republic;; d Department of Chemistry, University of Hradec Kralove, Hradec Kralove, Czech Republic

**Keywords:** Acetylcholinesterase, Alzheimer’s disease, donepezil, multi-target directed ligands, oxidative stress

## Abstract

Alzheimer’s disease is debilitating neurodegenerative disorder in the elderly. Current therapy relies on administration of acetylcholinesterase inhibitors (AChEIs) -donepezil, rivastigmine, galantamine, and *N*-methyl-d-aspartate receptor antagonist memantine. However, their therapeutic effect is only short-term and stabilizes cognitive functions for up to 2 years. Given this drawback together with other pathological hallmarks of the disease taken into consideration, novel approaches have recently emerged to better cope with AD onset or its progression. One such strategy implies broadening the biological profile of AChEIs into so-called multi-target directed ligands (MTDLs). In this review article, we made comprehensive literature survey emphasising on donepezil template which was structurally converted into plethora of MTLDs preserving anti-cholinesterase effect and, at the same time, escalating the anti-oxidant potential, which was reported as a crucial role in the pathogenesis of the Alzheimer’s disease.

## Graphical abstract

**Figure:**
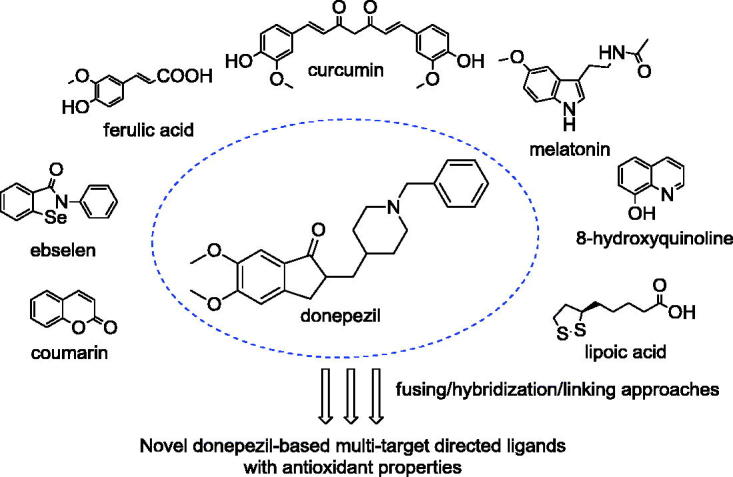

## 1. Introduction

Dementia is a chronic or progressive illness that is characterised by impaired cognitive capacity beyond what could be considered a consequence of normal aging. The most common form of it is the Alzheimer’s disease (AD) accompanied by the symptoms such as memory loss, difficulty in solving problems, comprehension, calculation and learning, disorientation, impaired learning ability etc^1^. According to the data from European Prevention of Alzheimer's Dementia, AD affects more than 40 million people worldwide and its prevalence is expected to double over the next 20 years. Moreover, AD is currently the fourth leading cause of death in people over 65 years old in the world, which makes it one of the major health, social, and economic concern of the society worldwide^2^ ^,^ ^3^.

Many pathological aspects of AD have been discovered in the course of over 100 years of research and observation of AD patients. One of the most important finding was the identification and functional characterisation of neurotransmitter acetylcholine (ACh)^4^. Indeed, the investigation of biopsy tissue and post-mortem brain tissues from AD patients showed reduced choline acetyltransferase activity, ACh synthesis, choline uptake and ACh release. Moreover, the impairment of cognitive functions is also associated with degeneration of cholinergic neurons and loss of cholinergic neurotransmission. In line with this discovery, novel therapeutic approaches were developed to “correct” or “compensate” for neurochemical alterations in the cholinergic system^5^. Besides, other disrupted neurotransmitter system attracted particular attention for targeting AD pathological pathways, such as *N*-methyl-d-aspartate (NMDA) receptors^6^ dopaminergic system^7^ serotoninergic system^8^ and others.

**Figure 1.**
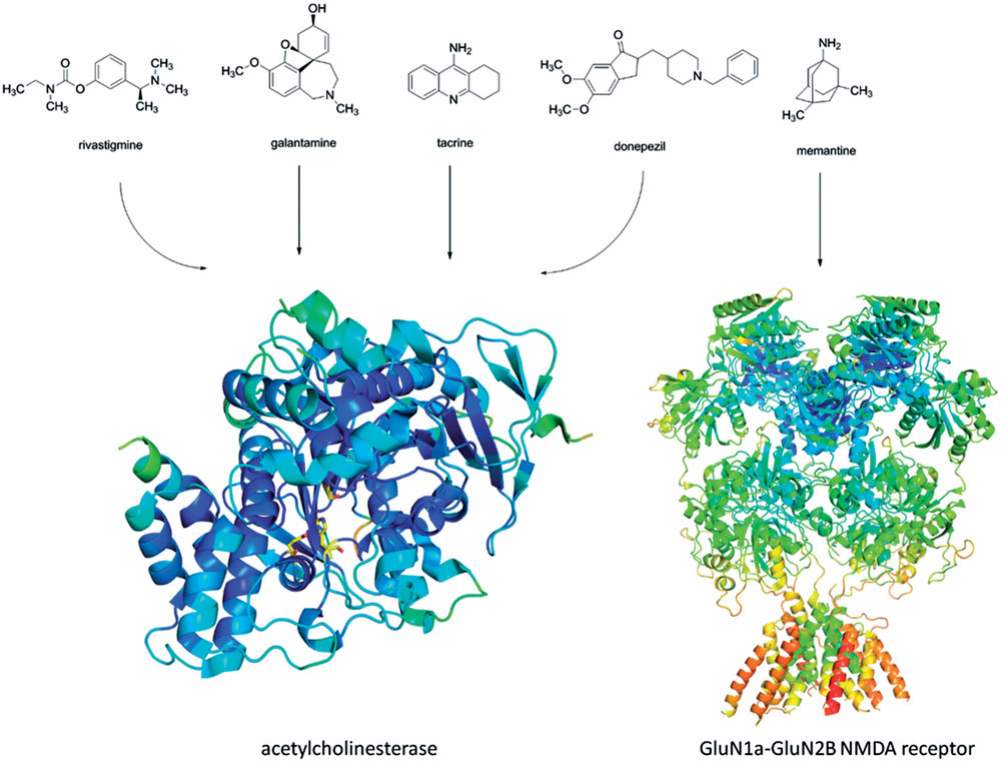
Currently used AChEIs donepezil, galantamine and rivastigmine. Tacrine is no longer approved for AD treatment. Memantine act as NMDA receptor antagonist. Structures of human AChE (PDB ID: 4EY7) and GluN1A-GluN2B NMDA receptor (PDB ID: 4PE5) were downloaded from Protein Data Banka (http://www.rcsb.org) and created with PyMol viewer 1.3.

Building on the aforementioned, currently approved drugs for AD rivastigmine, galantamine and donepezil, as well as discontinued tacrine (Figure 1), improve cholinergic neurotransmission by inhibition of acetylcholinesterase (AChE, E.C. 3.1.1.7), enzyme responsible for degradation of ACh^9^. While AChE predominates in the healthy brain, butyrylcholinesterase (BChE, E.C. 3.1.1.8) is considered to play a minor role in the regulation of synaptic ACh levels. This scenario is modified in the context of AD, as the activity of AChE remains unchanged and BChE activity progressively increases^10^. The AChE inhibitors (AChEIs) are used for symptomatic treatment of AD shedding light on the importance of AChE which still remain a highly viable classic target for development of new drug candidates^11^.

Another drug approved for AD is memantine (Figure 1) which acts as antagonist of NMDA receptor^12–14^. The connection between the constant stimulation of NMDA receptors and the cognitive deficits seen in AD is based primarily on the principle of excitotoxicity and the death of nerve cells is caused by chronic neuronal activation^15^.

Another two major players in the pathogenesis of AD postulated that formation of amyloid deposits and neurofibrillary tangles are responsible for the onset of the disease. Accordingly, the amyloid-β (Aβ) hypothesis implies that AD therapy should restore the Aβ homeostasis in the brain by altering the production and clearance of Aβ^16^. There are several strategies possible applied for the maintenance/reduction of Aβ levels in the brain: prevention or reduction of Aβ formation, removal of existing amyloid deposits through immunotherapy, prevention or reduction of Aβ aggregation, and enhancement of Aβ clearance^17^. Unfortunately, all efforts to develop Aβ-targeted therapies have failed to date. Moreover, recent studies have shown that both prevention of amyloid deposition and removal of amyloid do not, by themselves, lead to improved cognition in AD^18^. Another important therapeutic target involved in the pathogenesis of AD is the tau protein. Tau is ubiquitous in neurons and plays an important role in microtubule assembly and stabilisation of neuronal microtubule network^19^. In AD patients brain, high levels of hyperphosphorylated tau can be found intracellularly which lead to a generation of aberrant aggregates that are toxic to neurons^20^. None of the developed anti-tau drug candidates has also advanced to clinical practice to date. It has to be mentioned that current trend in drug design of novel AD drugs switches from anti-Aβ to anti-tau therapy^18^.

Oxidative stress is considered as one of the key players in the aetiology and progression of various neurodegenerative disorders. Abundant data suggests that oxidative stress may induce not only cellular damage, but also DNA repair system breakdown or mitochondrial malfunction. All of these events largely contribute to aging and neurodegeneration, a phenomena observed in AD or Parkinson’s diseases progression^21^. A group of reactive oxygen species (ROS) contain highly reactive and more or less short-lived molecules derived from oxygen. Among these, free radicals such as superoxide, hydroxyl radical, or hydrogen peroxide can be found being responsible for the cytotoxic effect^22^. Many biological systems have been implicated in ROS production like mitochondria, NADPH oxidases, xanthine oxidase, peroxisomes, or endoplasmatic reticulum^23^. Increased ROS levels may be down-regulated by several defence systems including antioxidant enzymes or endogenous small-molecule antioxidants (e.g. superoxide dismutase, glutathione peroxidase, catalase, peroxiredoxins, tri-peptide glutathione, vitamins E, and C)^24^. On the other hand, low levels of ROS have been shown to be involved in physiological processes like cellular signalling, pro-survival pathways or activation of transcription factors regulating cellular response to ROS^25^.

In general, oxidative stress could be regarded as imbalance between the generation of ROS and malfunction of natural antioxidant system. The depletion of antioxidant system or overproduction of ROS leads to pathological conditions denoted as oxidative stress. Under these conditions, brain is the most vulnerable organ in the body. Brain is characterised by the highest oxygen consumption and contains redox-active metals like iron or copper catalysing ROS formation. Furthermore, high levels of polyunsaturated fatty acids in the brain represent a good substrate for lipid peroxidation^26^.

The intertwined pathophysiological pathways of AD point to linkage between the Aβ accumulation and increased oxidative stress resulting in mitochondrial malfunction and energy collapse in the early stages of the disease^27^. Vice versa, oxidative stress may also escalate the production and aggregation of Aβ and mediate the phosphorylation of the tau protein^28^.

Administration of antioxidants for treating neurodegenerative disorders has brought contradictory results. On one side, the results from animal studies implied potential benefit of this therapy, on the other side clinical trials precluded to meet the expected outcomes and benefits^29^. Several explanations could be addressed to low efficacy of antioxidants in the treatment of AD. The reason for such behaviour can be found in insufficient dose of antioxidants, improper therapy management, or insufficient therapy duration. Other reasons could be also highlighted such as: (i) oxidative damage may not be the primary cause but the consequence of vicious cycle accompanying the pathophysiological processes of AD; (ii) single antioxidant may not be capable to sufficiently counteract the complex cascade of oxidative stress; (iii) earlier antioxidant administration would be better since most of the clinical trials focused on patients with advanced AD; (iv) patients should be more cautiously selected for clinical trials (i.e. patients with low levels of endogenous antioxidants are better responders)^30^ ^,^ ^31^.

Based on the above-mentioned reasons, multimodal approach combining antioxidant properties with other relevant targets involved in AD pathology is an interesting approach. In recent year, several articles describing the potential of novel compounds including antioxidant properties have been published^11^ ^,^ ^32^ ^,^ ^33^. This review article is focused on the drug design and development of novel multi-target directed ligands (MTDLs) amalgamating donepezil as core scaffold extending its biological profile primarily against oxidative stress.

## 2. Overview of *in vitro* methods for determination of antioxidant activity

Antioxidant capacity is connected with compounds able to protect a biological system against the damaging influence of ROS and reactive nitrogen species (RNS). The general capacity of the compound to scavenge ROS and/or RNS is called the total antioxidant activity (TAC)^34^. Nowadays, several chemical assays in combination with highly sensitive detection technologies are massively exploited for measurement of antioxidant activity of compounds through different mechanisms, including scavenging activity against specific radicals or ROS, hydrogen atom transfer (HAT), single electron transfer (ET), metal chelation, and reducing power^35–37^. *In vitro* methods for measuring antioxidant capability described in this section differ from each other in reaction condition and mechanism, substrate that is oxidised, target probe/species, and type of detection. These assays usually utilize a chemical system involving free radicals or other ROS (an oxidant), an oxidizable probe (for some assays is needless) and antioxidants.

HAT-based methods evaluate the capability of antioxidants to scavenge free radicals by hydrogen donation to form a stable compound. Most of these methods control competitive reaction kinetics and the effect is determined from the kinetic curves^38^. Among others, oxygen radical absorbance capacity (ORAC) method, total peroxyl radical-trapping antioxidant parameter (TRAP) assay using β-phycoerythrin or fluorescein as the fluorogenic probe and total oxyradical scavenging capacity (TOSC) test belong to HAT-based methods^39^ ^,^ ^40^. ET-based methods measure antioxidant capacity to reduce a probe (transfer one electron to reduce radicals, carbonyls, metals etc.). This process leads to colour change of the probe (an increase or decrease of the probe absorbance at a specific wavelength) after removing of an electron from the antioxidant. The stage of colour change depends on the concentration of antioxidant^40^. 2,2-Diphenyl-1-picrylhydrazyl (DPPH) radical scavenging, trolox equivalent antioxidant capacity (TEAC), 2,2-azinobis-(3-ethylbenzothiazoline-6-sulfonic) acid (ABTS) radical cation decolourisation, ferric reducing antioxidant power (FRAP) and cupric reducing antioxidant capacity (CUPRAC) assays are the most frequently used techniques of ET-based methods^41–45^. Although HAT and ET have different mechanism, both of them are able to reveal the nature of antioxidant profile in the tested compound.

### 2.1. TEAC assay

TEAC assay is a simple and convenient method for determination of TAC based on the ability of antioxidants to scavenge the stable ABTS radical cation (a blue–green chromophore with an absorption maximum wavelength at 734 nm). Potential antioxidants can neutralize the ABTS radical cation by either radical quenching through hydrogen atom donation or by direct reduction through electron donation. Thereby, the antioxidants decolorize ABTS radical cation and spectrophotometrically can be measured a decrease in absorbance (the loss of its colour). This depends on the intrinsic antioxidant activity, concentration sample and also reaction duration^35^ ^,^ ^44^ ^,^ ^46^. Trolox (6-hydroxy-2,5,7,8-tetramethylchroman-2-carboxyl acid, water soluble analogue of vitamin E) can be used as standard antioxidant and results of the experiments are usually expressed as Trolox equivalent^40^. This assay is usually classified as ET-based method.

### 2.2. ORAC assay

ORAC assay is relatively novel test tube analysis measuring the antioxidant scavenging activity against the peroxyl radical produced by a generator such as 2,2′-azobis(2-amidinopropane) dihydrochloride (AAPH), 2,2′-azobis(2,4-dimethylnaleronitrile) (AMVN) or 2,2-azobis(2-amidinopropane) hydrochloride (ABAP)^37^ ^,^ ^47^. The ability of potential antioxidant to slow or stop the radical reaction is observed. The peroxyl radical reacts with a fluorescent probe (fluorescein or β-phycoerythrin) resulting in decrease of fluorescence that can be recorded using spectrofluorimeter. Usually, Trolox is used as a reference and established ORAC values of the tested potential antioxidants are reported as Trolox equivalent. It is well-accepted that the higher ORAC values the better antioxidant ability of the compound. Considering the fact that ORAC assay measures hydrogen atom donating ability of antioxidants, it belongs to HAT-based methods^35^ ^,^ ^46^ ^,^ ^48^.

### 2.3. DPPH assay

DPPH radical scavenging assay is probably the most frequently used method. It is characterised as an ET-based method with HAT mechanism. Mechanistically, electron donation of antioxidants neutralizes DPPH radical. DPPH is a cell permeable and stable free radical with a deep violet colour (with an absorption maximum wavelength at around 520 nm). Obviously, reaction between DPPH and appropriate antioxidant is connected with its colour change by decreasing the absorbance of the system^35^ ^,^ ^36^ ^,^ ^38^.

### 2.4. FRAP assay

FRAP assay is a typical ET-based non-radical method depending on the reduction of ferric ion (Fe(III))-ligand complex by the antioxidants to navy blue colour ferrous (Fe(II)) complex. Antioxidant capacity is therefore defined as an increase of absorbance at around 593 nm and obtained results can be expressed as Fe^2+^ equivalents or relative to the antioxidant standards. Normally, 2,4,6-tripyridyl-s-triazine (TPTZ) is used in this assay as the iron-binding ligand and trolox or ascorbic acid as antioxidant standards^34^ ^,^ ^37^. In order to keep solubility of iron, FRAP assay is carried out under low acidic pH (pH = 3.6) which is far from the physiological pH values. Therefore, it is not possible to measure the antioxidants containing thiol groups^49^.

### 2.5. CUPRAC assay

CUPRAC assay was developed as a modification of FRAP assay replacing iron by copper as oxidant. This method determines the ability of antioxidants to reduce cupric ion (Cu(II))-ligand complex to cuprous ion (Cu(I)) complex (chromophore with absorption maximum at 450 nm). Neocuprine (2,9-dimethyl-1,10-phenanthroline) is usually used as ligand. In contrast with FRAP assay, CUPRAC assay is carried out at pH = 7 and therefore enlarge the versatility of this method to other substrates including thiol-containing compounds^49^.

## 3. Donepezil derivatives with antioxidant properties

### 3.1. Coumarin hybrids

Coumarin derivatives as AChEIs deserved closer attention because these types of naturally occurring as well as chemically developed compounds possess a wide range of pharmacological activities not only favourable for AD treatment but also for other maladies, and have been broadly reviewed^50^ ^,^ ^51^. Several studies indicating that coumarins are capable of inhibiting AChE by binding to its PAS have spurred the design and synthesis of novel coumarin derivatives as potent AChEIs^52–55^.

**Figure 2.**
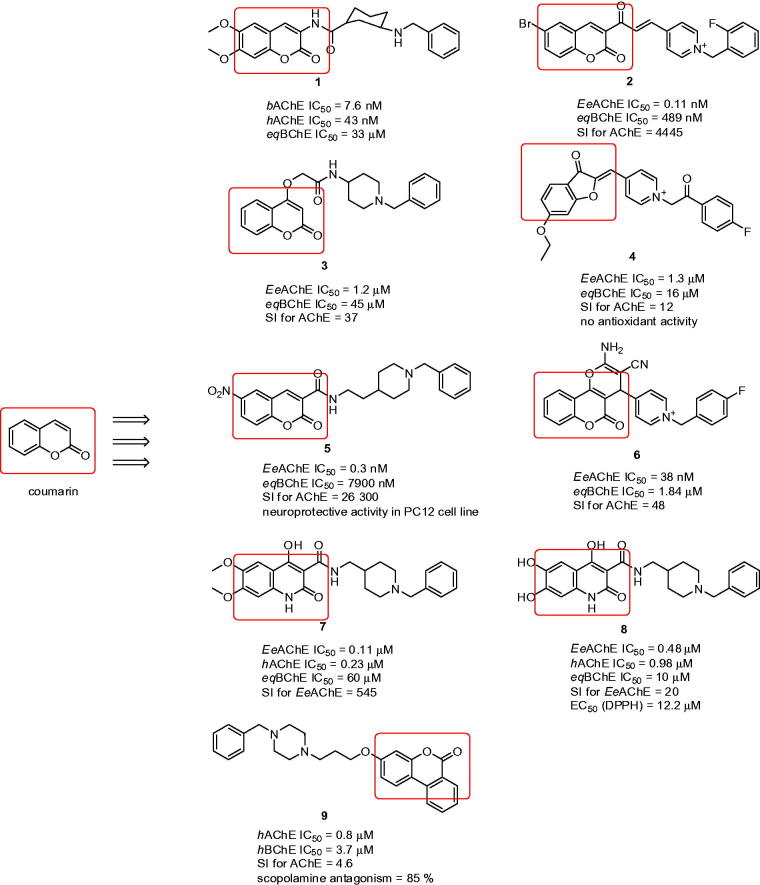
Donepezil-related coumarin derivatives.

A large number of coumarins endowed with high AChE inhibitory activities have been recently described^56–59^. 6,7-Dimethoxy-3-substituted coumarins linked to a benzylamine moiety placed at an appropriate distance from the heterocyclic ring were designed and reported (Figure 2)^60^. Inhibitory potency was chiefly affected by the distance between the amide attached to the coumarin moiety at the 3-position and the benzylamine fragment, with potency enhancement up to a trimethylene bridge. A small drop in inhibitory activity was observed when the amide group was replaced by an inverted amide group or isosteric ester. The same consideration as for donepezil claims that inhibitory activity declines when both methoxy groups attached to coumarin are removed. Compound **1**, a *cis*-3-amino-cyclohexanecarboxylic acid derivative, displayed AChE inhibitory potency comparable to that of the reference donepezil (**1**: *b*AChE IC_50_ = 7.6 nM; *h*AChE IC_50_ = 43 nM; *eq*BChE IC_50_ = 33 µM; Figure 2). The study confirmed a reversible, mixed-type model of inhibition indicating dual binding site character.

Prof Shafiee’s group reported a variety of synthetic approaches leading to biologically active coumarins^61–65^. In a preceding study, it was proved that the *N*-benzylpyridinium scaffold is required for superior activity^66^. First, coumarin ring derivatives connected to differently substituted *N-*benzylpyridinium moieties were investigated (Figure 2)^67^. The attachment of these moieties utilised an α,β-unsaturated carbon linker to confer conformational restriction, which made it possible to study substituent modifications regardless of any conformational alterations in the linker. The target compounds demonstrated AChE IC_50_ values in the range of moderate to outstanding, achieving picomolar concentrations with the most active derivative being **2** (*Ee*AChE IC_50_ = 0.11 nM; *eq*BChE IC_50_ = 489 nM; SI for AChE = 4445; Figure 2). SAR disclosed that the activity of these compounds mainly depends on the steric and electronic features of the substituents located on the benzyl moiety. Moreover, the activity was sensitive to the size of substituents at the *para* position of this moiety, with analogues containing bulky groups being weaker AChEIs. Coumarin ring modification was also performed, considering that insertion of a methoxy group (C-6) or bromine atom (C-8) is necessary for activity enhancement. In addition, two different methods, DPPH (1,1-diphenyl-1-picrylhydrazyl) and FRAP (ferric reducing antioxidant power) proved an absence of antioxidant ability compared to the reference compound (ascorbic acid). In the docking studies with *Tc*AChE, **2** revealed a similar arrangement to that of the reference drug donepezil. Importantly, docking studies confirmed the significance of positive charge within the *N*-benzylpyridinium moiety, as it can provide cation–π interaction with Tyr334 in the *Tc*AChE active site. This might be the reason for the 2-fold increased activity of **2** compared to donepezil.

A series of 4-hydroxycoumarin derivatives connected via an alkoxy amide spacer to *N*-phenylpiperazine or *N*-benzylpiperidine scaffolds was designed^68^. In the study, compound **3** bearing an *N*-(1-benzylpiperidin-4-yl)acetamide appendage displayed the highest inhibitory activity against *Ee*AChE (**3**: *Ee*AChE IC_50_ = 1.2 µM; *eq*BChE IC_50_ = 45 µM; SI for AChE = 38; Figure 2). SAR revealed that the anti-AChE activity of these compounds was influenced mainly by the type of the cyclic amine attached to the 2-oxo- or 4-oxoalkoxycoumarin skeleton. Docking studies performed on *Tc*AChE indicated that Phe330 is responsible for ligand recognition and trafficking by forming cation–π interaction with the *N*-benzylpiperidine moiety.

In another study, coumarin and 3-coumaranone derivatives encompassing the phenacyl pyridinium moiety were prepared^69^. In particular, compound **4** with a fluoro atom at the *para* position of the phenacyl moiety emerged as the most potent AChEI, with an IC_50_ value of 1.3 µM (for *Ee*AChE; *eq*BChE IC_50_ = 15.8 µM; SI for AChE = 12; Figure 2). Based on kinetic studies, the mode of inhibition indicated mixed type. Compounds in this subset were also tested for their antioxidant properties using FRAP assay. However, none of the compounds displayed significant antioxidant activity in comparison with ascorbic acid as reference, except the analogues containing a methoxy group or catechol group on the phenacyl moiety. Moreover, all compounds in the series exhibited desirable ADME properties with the ability to penetrate BBB, having polar surface area (PSA) less than^70^ 60–70 Å^2^.

Two structural motifs combining *N*-benzylpiperidine with coumarin that are connected either via a carboxamide or *N*-ethylcarboxamide linker were designed and synthesised^71^. Compounds containing the latter linker were more active than their counterparts with carboxamide linker. The most active derivative was found to be **5**, bearing a nitro group at position 6 of the coumarin ring, which was 46-fold more potent than donepezil (*Ee*AChE IC_50_ = 0.3 nM; *eq*BChE IC_50_ = 7900 nM; SI for AChE = 26 300; Figure 2). Several compounds were determined for cell viability using MTT assay, which indicated no toxicity at concentrations of 1–100 µM. Neuroprotective properties against H_2_O_2_ in differentiated PC12 cells were evaluated within the 1–100 µM range, verifying that pre-treatment with these compounds significantly protected neurons against cell death at all the compound’s tested concentrations. The docking studies revealed that the higher flexibility of the *N*-ethylcarboxamide linker in compound **5** might lead to more facile accommodation of the compound into the active site, with better dual binding site inhibition of AChE. This was confirmed by kinetic analysis, which assigned a mixed type mode of inhibition.

Fused coumarins, namely 5-oxo-4,5-dihydropyrano[3,2-*c*]chromene derivatives linked to an *N*-benzylpyridinium scaffold were developed as AChEIs^72^. The most potent compound was found in the 4-pyridinium series (**6**: *Ee*AChE IC_50_ = 38 nM; *eq*BChE IC_50_ = 1.84 µM; SI for AChE = 48; Figure 2), while the 3-pyridinium series exhibited still good, but rather lower anti-AChE activity in comparison to the 4-substituted series. The SAR including molecular modelling studies disclosed that the presence of electron-withdrawing groups such as fluoro- or chloro- on the appropriate position of the benzylic pendent could lead to reinforcing of π–π interaction with some aromatic residues in the PAS of AChE. Noteworthy, the pyranochromene moiety is oriented within CAS, while the benzyl moiety provides interaction with PAS residues. Such dual binding site character is in agreement with the data obtained from kinetic analysis pointing to mixed type inhibition. Based on the predicted values of BBB penetration, all compounds in the series might be able to permeate into the CNS. Moreover, calculated LC_50_ values (50% of lethal concentration) indicated that these compounds might show neither acute toxicity nor mutagenic effect, the latter according to AMES test data. Finally, all compounds fulfilled the Lipinski criteria of drug likeness. More recently other authors have extended their study into hybridising the tetrahydroaminoquinoline and coumarin scaffolds. However, this combination does not contain any structural feature related to donepezil^73^.

Quinolone–benzylpiperidine ChEIs with high radical scavenging activities were established by Pudlo et al.^74^ Quinolone-containing compounds exhibited a wide variety of biological activities, one of which is ROS scavenging ability and in some broad context can be considered as coumarin derivatives^75^ ^,^ ^76^. The described compounds showed mostly moderate selectivity for *Ee*AChE over *eq*BChE. *In vitro* experiments singled out to two compounds (**7**: *Ee*AChE IC_50_ = 0.11 µM; *h*AChE IC_50_ = 0.23 µM *eq*BChE IC_50_ = 60 µM; SI for *Ee*AChE = 545; and **8**: *Ee*AChE IC_50_ = 0.48 µM; *h*AChE IC_50_ = 0.98 µM *eq*BChE IC_50_ = 10 µM; SI for *Ee*AChE = 20; Figure 2) as being the best in term of ChE inhibition. The SAR in the series can be enlightened as follows: (i) a methylene linkage between quinolone-carboxamide and *N*-benzylpiperidine improved anti-AChE potency; (ii) *N*-substituted quinolone derivatives displayed reduced activity and solubility compared to unsubstituted compounds; (iii) a catechol moiety on the quinolone ring conferred the best radical-scavenging activity. With regard to antioxidant properties, the EC_50_ (DPPH assay) for compound **8** (EC_50_ (DPPH) = 12.2 µM) was found to be similar to that of standard quercetin and 2-fold higher than for curcumin. The docking studies demonstrated that a quinolone-attached catechol moiety is involved in interactions with AChE in a similar way to the dimethoxyindanone motif from donepezil with *N-*benzylpiperidine being stacked against Trp286 (*h*AChE) in the CAS.

Ellagitannins constitute one of the major classes of polyphenolic natural products. Their chemical structures are basically composed of a central sugar core, typically d-glucopyranose, to which are esterified gallic acid (i.e. 3,4,5-trihydroxybenzoic acid) units^77^. Ellagitannins are macromolecules with no bioavailability; however, they can be fully converted in the human gastrointestinal flora to urolithins (i.e. hydroxylated 6*H*-benzo[*c*]chromen-6-one derivatives), which do not provide any AChE/BChE inhibition activity^78^. In an attempt to develop urolithin-related compounds endowed with anti-ChE properties, rivastigmine-like and donepezil-like analogues were designed employing the 6*H*-benzo[*c*]chromen-6-one moiety present in urolithins^79^. Since central action is required in the treatment of AD, more saturated 7,8,9,10-tetrahydrobenzo[*c*]chromen-6-one derivatives related to tetrahydrocannabinol were also designed with regard to their potential to penetrate the CNS. Focused on donepezil-related analogues, three different regions were included into the SAR study: (i) the saturation level of the benzo[*c*]chromen-6-one moiety; (ii) the carbon chain length bridging the benzo[*c*]chromen-6-one system to the amine group; (iii) the various amine substitutions. All members in this category displayed selective pattern behaviour for AChE, but were far behind donepezil or galantamine. The most active derivative also displayed comparable activity to that of donepezil and rivastigmine in the scopolamine induced passive avoidance test (**9**: *h*AChE IC_50_ = 0.8 µM; *h*BChE IC_50_ = 3.7 µM; SI for AChE = 4.6; scopolamine antagonism = 85%; Figure 2).

### 3.2. Ferulic acid and curcumin hybrids

**Figure 3.**
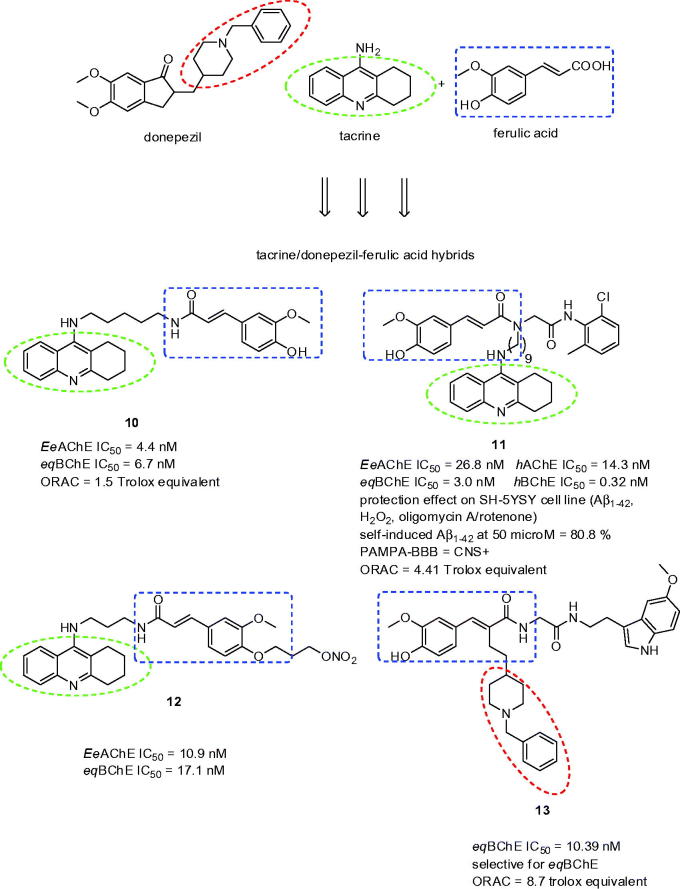
Ferulic acid hybrids possessing antioxidant and anticholinesteratic properties.

Ferulic acid is a natural phenolic acid possessing potent anti-oxidative and anti-inflammatory activities^80^. With these precedents in mind, ferulic acid has become largely exploited scaffold for design of novel multipotent compounds like tacrine-ferulic acid hybrids with potential implication for AD treatment^81–83^. Examples of tacrine-ferulic acid hybrids are shown in Figure 3 (compounds **10**, **11**, and **12**). Inspired by these studies, twelve novel donepezil-ferulic acid hybrids were prepared as potent non-selective *Ee*AChE and *eq*BChE inhibitors using one-pot Ugi reaction^84^. In general, all the compounds exerted balanced inhibition profile being less active *Ee*AChE inhibitors and more potent *eq*BChE inhibitors related to donepezil. In addition, these hybrids displayed stronger antioxidant power than the reference compounds ferulic acid and melatonin. Based on the preliminary results, the study highlighted *eq*BChE selective derivative **13** (*eq*BChE IC_50_ = 10.39 nM; ORAC = 8.7 Trolox equivalent; Figure 3).

**Figure 4.**
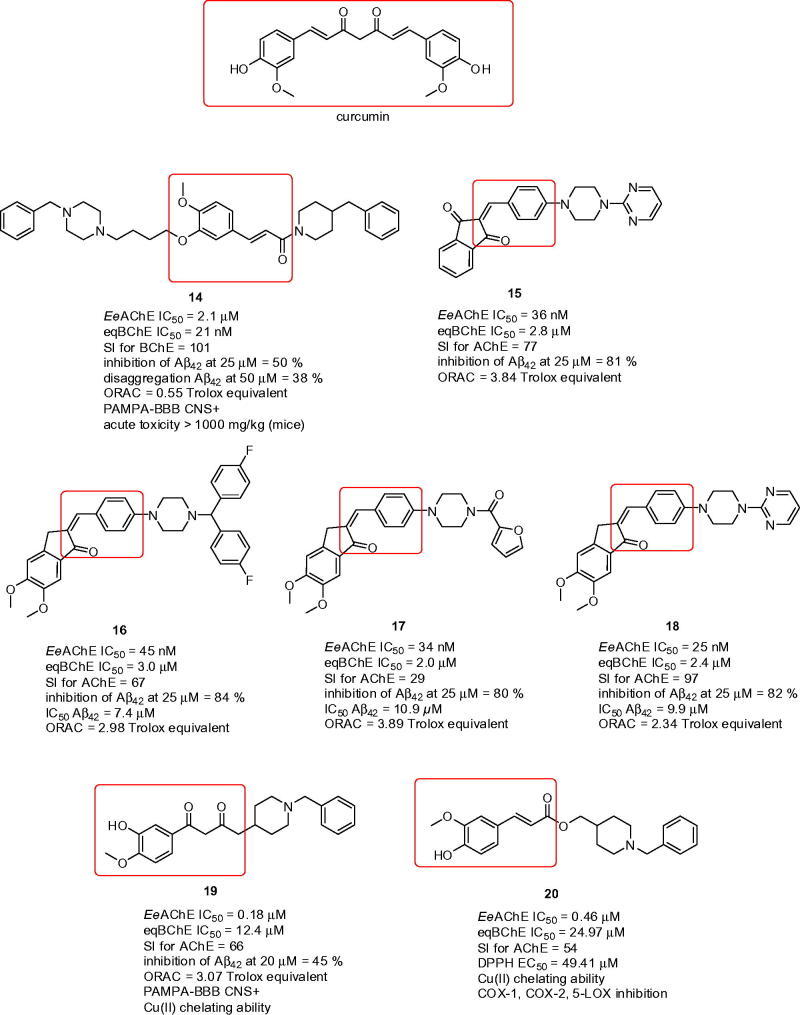
Curcumin-based hybrids with antioxidant properties.

Ferulic acid-*O*-alkylamines can be classified as MTDLs combining ferulic acid with *N*-benzylpiperidine fragment into single molecule^85^. The pharmacological assessment of this subset consisted of screening inhibition against *Ee*AChE, *eq*BChE, where some of the compounds were reevaluated on *rat*AChE, *rat*BChE, *h*AChE, and *h*BChE. Next, the Aβ_42_ self-aggregation and disaggregation tests, antioxidant properties assessment, cytotoxicity, cell protective effects on H_2_O_2_ induced PC12 cell injury, prediction of BBB permeation were performed for the selected compound **14** (*Ee*AChE IC_50_ = 2.1 µM; *eq*BChE IC_50_ = 21 nM; SI for BChE = 101; *rat*AChE IC_50_ = 1.8 µM; *rat*BChE IC_50_ = 8.6 µM; *h*AChE IC_50_ = 3.8 µM; *h*BChE IC_50_ = 70 nM; inhibition of Aβ_42_ at 25 µM = 50%; disaggregation Aβ_42_ at 50 µM = 38%; ORAC = 0.55 Trolox equivalent, Figure 4) that was chosen based upon its anti-cholinergic properties. Moreover, the study was supplemented by *in vivo* data counting acute toxicity and the step-down passive avoidance tests. The latter was aimed to observe whether **14** can improve the contextual memory in scopolamine-induced mice. Indeed, **14** exerted improvement in cognitive decline when compared with the control group.

Curcumin (Figure 4) is another compound of natural origin derived from *Curcuma longa L.* (*Zingiberaceae*)^86^. Its chemical structure can be regarded as biosynthetic dimer related to phenylalanine and cinnamic acid, respectively^87^. In traditional medicine, dried turmeric root was a remedy in several pathological conditions, among others skin diseases, wounds, rheumatisms, asthma, allergies, sinusitis, hepatic disorders, intestinal worms, and generic inflammation^88^. The neuroprotective potential of curcumin or mixture of curcumin derivatives, denoted as curcuminoids, has been confirmed not only in *in vitro* but also *in vivo* by attenuating inflammation and microglia activation in AD mouse model^89^. Curcumin have attracted researchers attention since it has been found to exert radical scavenging and iron chelation properties in brain tissue homogenate^90^. Apart from that, it displayed inhibition ability of AChE, protection in PC12 cell line against Aβ-induced damage and also direct inhibition of Aβ_42_ fibrillogenesis^91^ ^,^ ^92^. All these protective characteristic either of curcumin or curcuminoids are amplified via its anti-inflammatory action and downregulation of Aβ production through BACE1 expression^93^ ^,^ ^94^. Furthermore, curcumin can also modulate the phosphorylation of tau protein impeding the formation of neurofibrillary tangles (NFTs)^95^.

Taken the aforementioned precedents together, curcumin have become an important building block in designing novel compounds with potential implication in neurodegenerative diseases. One such work rationally designed a series of 2-(4-(4-substituted piperazin-1-yl)benzylidene)-1*H*-indene-1,3(2*H*)-diones comprising indanone moiety, curcumin fragment and piperazine six-membered ring into one molecule^96^. Multipotent profile of these compounds was confirmed in several experiments. Indeed, novel hybrids displayed micromolar to two-digit nanomolar inhibition potency against *Ee*AChE and one-digit micromolar to sub-micromolar *eq*BChE inhibition ability. Accordingly, the hybrids resulted in preferential AChE inhibition over BChE. With regard to AChE affinity, phenyl ring substitution favoured *para* substituted electron-withdrawing groups (EWGs) such as trifluoromethyl group over unsubstituted, electron-donating groups (EDGs) or disubstituted ones. Improvement in anti-cholinestrease activity was reached by replacing phenyl moiety with other heterocycles. In this context, remarkable activity was demonstrated by pyrimidine derivative **15** (*Ee*AChE IC_50_ = 36 nM; *eq*BChE IC_50_ = 2.8 µM; SI for AChE = 77; inhibition of Aβ_42_ at 25 µM = 81%; ORAC = 3.84 Trolox equivalent; Figure 4) being slightly more potent than donepezil under experimental conditions. Kinetic experiments indicated mixed-type pattern inhibition of **15**. Heterocyclic ring attachment to piperazine moiety also significantly improved inhibition of Aβ_42_ self-aggregation. The latter was confirmed by Thioflavin–T (ThT) fluorescence assay as well as by transmission electron microscopy either through direct interaction or by blocking the PAS of AChE. Antioxidant capacity, established by ORAC-FL and H_2_O_2_-induced SH-SY5Y cell-based stress assays, ranged from 0.55 to 3.52 of Trolox value throughout the series. This is consistent with the design where amalgamating curcumin scaffold into novel hybrids broadens the compounds profile. Neuroprotective profile was also elucidated using the model of H_2_O_2_- or Aβ-induced neurotoxicity.

Very close strategy was applied in another family combining piperazine, 5,6-dimethoxy-2,3-dihydro-1*H*-inden-1-one moiety and curcumin fragment^97^. The preparation exploited Knoevenagel condensation to form novel (*E*)-2-(4-(4-(substitued)piperazin-1-yl)benzylidene)-5,6-dimethoxy-2,3-dihydro-1*H*-inden-1-one derivatives. Given the structural similarity to previous subset, the inhibition potency also ranged from micromolar to low-nanomolar and micromolar to low-micromolar scales against *Ee*AChE and *eq*BChE, respectively. The structure–activity relationship (SAR) analysis disclosed that activity is more pronounced for *para*-substituted phenyl ring even by introduction of electron rich moieties like methyl, 2,4-dimethyl or 4-methoxy groups. The introduction of linker between phenyl ring and adjacent piperazine had detrimental effect on ChE affinity. Again, phenyl replacement by heterocycles like furoyl, pyridine, or pyrimidine yielded in notable activity enhancement. Based on these results, three compounds emerged as the new leads with comparable inhibition ability to donepezil (**16**: *Ee*AChE IC_50_ = 45 nM; *eq*BChE IC_50_ = 3.0 µM; SI for AChE = 67; inhibition of Aβ_42_ at 25 µM = 84%; Aβ_42_ IC_50_ = 7.4 µM; ORAC = 2.98 Trolox equivalent; **17**: *Ee*AChE IC_50_ = 34 nM; *eq*BChE IC_50_ = 2.0 µM; SI for AChE = 29; inhibition of Aβ_42_ at 25 µM = 80%; Aβ_42_ IC_50_ = 10.9 µM; ORAC = 3.89 Trolox equivalent; **18**: *Ee*AChE IC_50_ = 25 nM; *eq*BChE IC_50_ = 2.4 µM; SI for AChE = 97; inhibition of Aβ_42_ at 25 µM = 82%; Aβ_42_ IC_50_ = 9.9 µM; ORAC = 2.34 Trolox equivalent Figure 4).

Fusing β-diketonate-phenyl substituted analogs with *N*-benzylpiperidine provided novel class of MTDLs targeting *Ee*AChE, *eq*BChE, metal imbalance, oxidative stress pathway and Aβ_42_ self-aggregation process^98^. *N*-Benzylpiperidine attachment was conjugated either via the α-carbon between two ketone groups or via terminal carbon of curcumin residue appendage. The length of the tether between basic scaffolds played crucial role, the highest inhibitory potency was coined to two methylene units. 3-Hydroxy-4-methoxy disubstitution of curcumin phenyl ring emerged as the most favourable in terms of prevailing high AChE inhibition and escalated selectivity. Antioxidant activity was associated with free phenolic group in phenyl of curcumin skeleton. The most potent hybrid **19** (*Ee*AChE IC_50_ = 0.18 µM; *eq*BChE IC_50_ = 12.4 µM; SI for AChE = 66; inhibition of Aβ_42_ at 20 µM = 45%; ORAC = 3.07 Trolox equivalent; Figure 4) under the study also revealed the highest inhibition rate of Aβ_42_ self-aggregation by 45% at 20 µM. In concept with the design of these templates, kinetic characterisation of **19** revealed dual binding site character. This data was consistent with *in silico* experiments, however proposing that *N*-benzylpiperidine moiety of donepezil is oriented outwards the active gorge giving interaction with Trp279 from the PAS. To supplement therapeutic profile, the metal-chelating properties of **19** towards selected biometals like Cu(II), Fe (II), and Zn (II), were inspected. The latter might prevent from the slow accumulation of Aβ_42_ and thus impede its precipitation and crosslinking^99^. Indeed, compound **19** displayed 1:1 stoichiometry with Cu(II) thus could provide positive outcome likewise clioquinol or PBT2^100^ ^,^ ^101^.

Feruloyl-donepezil hybrids were developed as another example of novel and potent MTDLs^102^. Variation of the substituent groups in the aromatic region of *N*-benzylpiperidine derived from donepezil as well as aromatic part of feruloyl were explored in the effort to identify novel drug candidate. Unsubstituted *N*-benzylpiperidine derivatives showed sub-micromolar *Ee*AChE inhibition activity and significant selectivity over *eq*BChE (SI >50). In this regard, the study highlighted the hybrid **20** (*Ee*AChE IC_50_ = 0.46 µM; *eq*BChE IC_50_ = 24.97 µM; SI for AChE = 54; DPPH EC_50_ = 49.41 µM; Figure 4) being non-competitive AChE inhibitor with a *K*_i_ value of 1.04 µM. Almost all of the derivatives were effective in scavenging free radicals, however, not reaching the activity of ferulic acid itself as parent compound. This results might be sufficiently explained by analogy with the effect of *para*-hydroxy substituted cinnamic acids, in which the oxygen atom of the hydroxyl group is able to share a positive charge and thereby increase radical stabilisation through conjugation extension^103^. To counteract the intracellular ROS formation induced by H_2_O_2_ in SH-SY5Y cells was measured for **20** suggesting also its high effectiveness in the cellular model. Moreover, **20** selectively chelated Cu(II) and Fe(II) but not Fe(III) and Zn(II) thus broadening its biological profile. Neuroprotective profile of **20** was also proved when neurotoxicity was elicited by Aβ_42_ oligomers using SH-SY5Y cells. Given the fact that curcumin mediates its anti-inflammatory effects, among others, via inhibition of cyclooxygenase 1 (COX-1), cyclooxygenase 2 (COX-2), and 5-lypoxygenase (5-LOX) authors were also encouraged to see the effect of **20** towards these enzymes. Indeed, **20** decreased the activity of COX-1, COX-2, and 5-LOX by 40, 27, and 30%, respectively, measured via immunosorbent assay in mice serum. To confirm the latter *in vivo*, the carrageenan-induced paw oedema assay was conducted pointing out to the same mechanism of action as indomethacin.

### 3.3. Selenium derivatives with antioxidant properties

**Figure 5.**
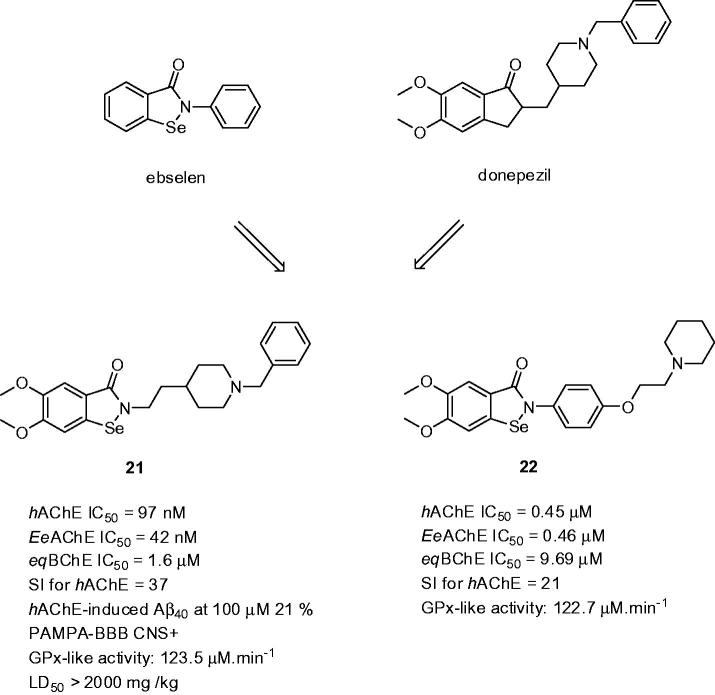
“Selenpezil” derivatives **21** and **22** based on fused ebselen with donepezil.

Selenium (Se) is an essential trace mineral nutrient with multiple roles in the growth and function of living animal cells, also providing protection against free radical-induced cell damage^104^. Several studies have indicated that not only the Se levels decrease with age, which may be in contrast with in the progression of AD^105^. Ebselen [2-phenyl-1,2-benzisoselenazol-3(2*H*)-one; Figure 5] is a lipid-soluble derivative mimicking glutathione peroxidase in the way that it is able to protect cells by catalysing the reduction of peroxides by glutathione^106^. Moreover, ebselen exerts anti-inflammatory activity and is able to inhibit iron-induced tau phosphorylation, both actions required for AD treatment^107^. Fusion strategy of ebselen and donepezil led to the discovery of so-called “selenpezil” compounds (Figure 5)^108^. SAR analysis revealed importance of the methoxy groups in the benzoselenazol-3(2*H*)-one moiety for inhibition activity. The length of the linker between the benzoselenazol-3(2*H*)-one moiety and piperidine ring is also important for maintaining activity, with a two-carbon spacer exhibiting the highest inhibition potency. Several biological assays highlighted compound **21**, which was able to inhibit AChE-induced Aβ_40_ aggregation (**21**: *h*AChE IC_50_ = 97 nM; *Ee*AChE IC_50_ = 42 nM; *eq*BChE IC_50_ = 1.6 µM; SI for AChE = 37; *h*AChE-induced Aβ_40_ at 100 µM 21%; Figure 5). Compound **21** exerted glutathione peroxidase-like activity, inferring the compound’s detoxification of hydroperoxides. Antioxidant properties were further confirmed indicating its scavenging ability for H_2_O_2_ and peroxynitrite removal. Based on PAMPA-BBB assay, the compound could be considered as centrally active. Further *in vivo* results with mice showed no acute toxicity or mortality, even after administration of high doses (2000 mg/kg of **21**). Other glutathione-peroxidase (GPx) mimics based on ebselen were reported more recently^109^. As shown in Figure 5 for the most active derivative **22**, the substitution at C-4 of the ebselen phenyl ring was carried out with attachment of different tertiary amines (pyrrolidine, piperidine, and *N,N*-dimethyl-1-phenylmethanamine) through an ether-methylene chain or methylene chain of variable length. Compound **22** possesses good inhibitory potency against ChE (*h*AChE IC_50_ = 0.45 µM; *Ee*AChE IC_50_ = 0.46 µM; *eq*BChE IC_50_ = 9.7 µM; SI for AChE = 21; Figure 5) and antioxidant activity against H_2_O_2_ *in vitro*.

### 3.4. Donepezil-like analogues with altered indane motif

**Figure 6.**
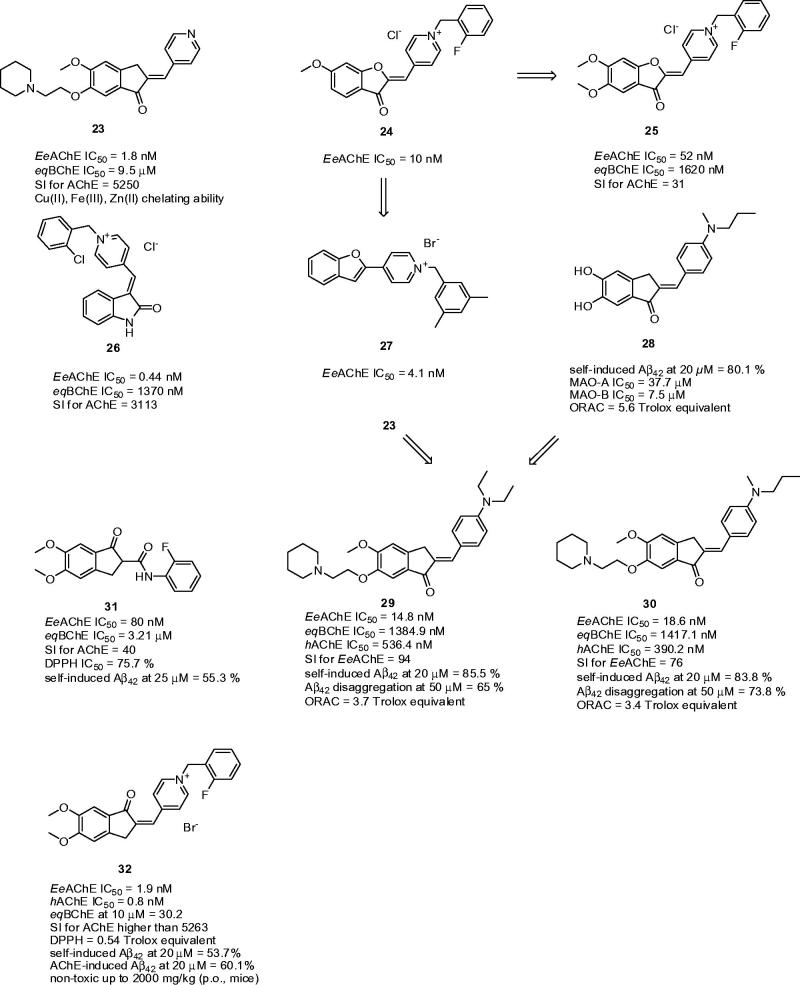
Indane-based ChEIs **23–32**.

New pyridinylmethylene-indanone derivatives endowed with metal-chelating and anti-cholinesterase properties were recently developed by the Meng group^110^. Different amines (piperidinyl, pyrrolidinyl, or *N,N-*diethylamine) were introduced into the C-6 position of the indanone moiety attached by a polymethylene bridge of variable size. The study highlighted the analogues with a piperidinyl substituent and a short spacer (two-carbon linker) as the most active ones. With regard to the aurone scaffold, a double bond was crucial for preservation of high affinity to *Ee*AChE. Pyridinylmethylene-indanone derivatives exerted rather moderate potency against *eq*BChE, thus being very selective for AChE. Amongst them, compound **23** exhibited the highest AChE inhibitory activity, being 47-fold more potent than tacrine and 14-fold more active than donepezil (*Ee*AChE IC_50_ = 1.8 nM; *eq*BChE IC_50_ = 9.5 µM; SI for AChE = 5250; Figure 6). Kinetic studies indicated a mixed type of inhibition. Moreover, **23** showed chelation ability towards biometals such as Cu(II), Fe(III) and Zn(II), suggesting its multifunctionality in AD treatment. In pursuit of promising results with aurone derivatives, the Nadri group prepared and evaluated a novel series of pyridinium analogs maintaining the planar conformation of the aurone ring^66^. The SAR indicated that introduction of a fluorine atom at position C-2 or C-4 of the benzyl fragment led to increased anti-AChE activity, which is in agreement with data previously reported. Furthermore, it was observed that a methoxy group at C-6 of the benzofuranone moiety delivered higher activity than the ethoxy and propoxy analogues. In this subset, compound **24** was highlighted as the most potent against *Ee*AChE (*Ee*AChE IC_50_ = 10 nM; Figure 6). Subsequently this pyridinium series was enlarged by using the more polar 5,6-dimethoxybenzofuranone scaffold, producing results very similar to those of **24** ^111^. As anticipated, compound **25** bearing a fluorine atom at position C-2 of the benzyl moiety showed the highest anti-AChE potency, being less active than its 6-methoxy counterpart **24** (**25**: *Ee*AChE IC_50_ = 52 nM; *eq*BChE IC_50_ = 1620 nM; SI for AChE = 31; Figure 6). Inspired by these findings, Shafiee and co-workers designed and synthesised indoline-based AChEIs, also bearing the benzylpyridinium moiety^112^. Screening assay revealed very potent inhibitory activities (*Ee*AChE IC_50_ = 0.44–12.8 nM) exceeding the standard donepezil. Of these, the 2-chorobenzyl derivative (**26**: *Ee*AChE IC_50_ = 0.44 nM; *eq*BChE IC_50_ = 1370 nM; SI for AChE = 3113; Figure 6) became the most potent compound against AChE. The SAR disclosed that substitution of the C-2 or C-3 position of the *N*-benzyl pendent by a halogen, methyl or methoxy group significantly improved the anti-AChE activity, while substitution at C-4 of the *N*-benzyl group diminished the activity. The observed IC_50_ values for *eq*BChE displayed that almost all compounds in the series exceeded the activity of donepezil towards this enzyme. Likewise, substitution of the *N*-benzyl moiety proved to have a positive effect on anti-BChE activity. The docking studies indicated three types of interactions involved in the attachment of ligand to *Tc*AChE, such as hydrophobic interaction, π–π interaction and cation–π interaction. The latter is responsible for interaction between the charged nitrogen of **26** and the mid-gorge site comprising Phe330, thus facilitating ligand recognition. Kinetic analysis performed on **26** was in agreement with the proposed binding mode of AChE displaying mixed-type inhibition (*K*_i_ = 1.14 nM). The prediction of ADMET analysis using web-based application suggested a centrally active character for these compounds, low acute toxicity according to the calculated LC_50_ values, and no mutagenic effect according to the AMES test^113^. Another survey focused on benzofuran-derived *N-*benzylpyridinium bromides highlighted derivative **27** as being 7-fold more potent than donepezil (**27**: *Ee*AChE IC_50_ = 4.1 nM; Figure 6)^114^. Docking studies with **27** revealed that the *N*-benzylpyridinium part of **27** was situated around Trp84, in the vicinity of the catalytic site. The positively charged nitrogen contributes in formation of a π–cation interaction with aromatic residues (Phe330 and Tyr334) at the mid-gorge recognition site.

Benzylideneindanone derivatives showed an interesting and broad biological profile potentially applicable for AD treatment^115^. In particular, compound **28** (Figure 6) gave the greatest inhibitory potency toward self-induced Aβ_42_ aggregation (80.1% at 20 µM). Moreover, **28** was an excellent antioxidant (ORAC = 5.60 Trolox equivalent) and monoamine oxidase (MAO)-A/B inhibitor (MAO-A IC_50_ = 37.7 µM; MAO-B IC_50_ = 7.5 µM). **28** was also found to be a good biometal chelator; it inhibited Cu(II)-induced Aβ aggregation and it could also disassemble well-structured Aβ fibrils. Another series of indanone derivatives combining the excellent AChE inhibitory profile of **23** with the anti-Aβ aggregation properties of **28** were further developed^116^. Indeed, the novel hybrids were potent inhibitors of *Ee*AChE with IC_50_ values in the nanomolar range. It is also important to note that the compounds showed weaker inhibition activities against *h*AChE and they also demonstrated slightly higher inhibitory potency against *eq*BChE than donepezil. The two most active compounds **29** (*Ee*AChE IC_50_ = 14.8 nM; *eq*BChE IC_50_ = 1384.9 nM; *h*AChE IC_50_ = 536.4 nM; SI for *Ee*AChE = 94; Figure 6) and **30** (*Ee*AChE IC_50_ = 18.6 nM; *eq*BChE IC_50_ = 1417.1 nM; *h*AChE IC_50_ = 390.2 nM; SI for *Ee*AChE = 76; Figure 6) were used for other biological investigation. Kinetic studies with **29** indicated a mixed type inhibition, and hence indanone members could simultaneously target PAS and CAS of AChE. All the derivatives demonstrated excellent antioxidant activity with ORAC values of 1.67–3.88 Trolox equivalents. Compared to curcumin, both compounds exerted markedly higher inhibitory activity in self-mediated Aβ_42_ aggregation assay (at 20 µM 52.1%, 85.5%, and 83.8% for curcumin, **29** and **30**, respectively). The number of Aβ fibrils was significantly decreased after their incubation with **29** and **30** (at 50 µM 65.0% and 73.8% disaggregation rates for **29** and **30**, respectively). Based on PAMPA-BBB assay, it can be predicted that **29** and **30** can permeate BBB.

Modified donepezil analogues bearing a secondary aromatic amide moiety displayed interesting pharmacological profile including inhibitory effects on ChEs and Aβ_42_ aggregation with antioxidant and metal chelating abilities^117^. 5,6-Dimethoxy-indanone ring from donepezil was maintained attaching *ortho*-, *meta*-, or *para*-substituted secondary aromatic amines via carbonyl linker. All the compounds revealed selective AChE inhibition potency ranging from 0.08 to 0.92 µM concentrations observing that higher activity is bound to EWG-containing substituents on aromatic moiety. The latter is consistent with previous studies^118^ ^,^ ^119^. The highest inhibition activity reached *p-*fluoro substituted benzamide **31** being mixed-type AChE inhibitor (**31**: *Ee*AChE IC_50_ = 80 nM; *eq*BChE IC_50_ = 3.21 µM; SI for AChE = 40; Figure 6). All the *meta*- and *para*-substituted compounds conferred more positive contribution toward BChE inhibition than *ortho*-substituted compounds. When incubated with Aβ_42_, **31** displayed 55.3% propensity to inhibit Aβ self-aggregation at 25 µM. In general, all the hybrids in the subset demonstrated both moderate antioxidant properties and ability to complex Zn(II). All these features are presumably coined to the presence of beta-keto-amide moiety in the structure.

*N*-Benzylpyridinium with 5,6-dimethoxy-indanone were constructed primarily targeting ChEs, oxidative stress and Aβ pathological pathways^120^. The similar pattern for AChE inhibition was observed for **31** as well as for **32** bearing 2-fluoro substituent. The inhibitory activity against AChE was 21-fold higher than for donepezil with the negligible BChE inhibitory activity and significant AChE selectivity (**32**: *Ee*AChE IC_50_ = 1.9 nM; *h*AChE IC_50_ = 0.8 nM*; eq*BChE at 10 µM = 30.2%; SI for *Ee*AChE >5263, Figure 6). In the kinetic study, **32** was found to be mixed-type AChE inhibitor. **32** is potentially able to counteract Aβ pathological pathway via two distinct mechanisms: (i) by direct interaction with Aβ_42_ (53.7% inhibition at 20 µM) and (ii) by interaction with PAS of AChE to decrease the AChE-accelerated Aβ aggregation (60.1% inhibition at 20 µM). In general, all the pyridinium salts were moderate antioxidants. With this respect, **32** yielded as the most potent free radical scavenger possessing DPPH value 0.54 of Trolox equivalent. Moreover, **32** increased cell viability of PC12 cell line in the presence of both Aβ and H_2_O_2_ insults indicating its neuroprotective profile. Finally, **32** can be classified as centrally active based on the observations from the PAMPA-BBB assay. No acute toxicity was observed when orally administered to mice at doses up to 2000 mg/kg.

**Figure 7.**
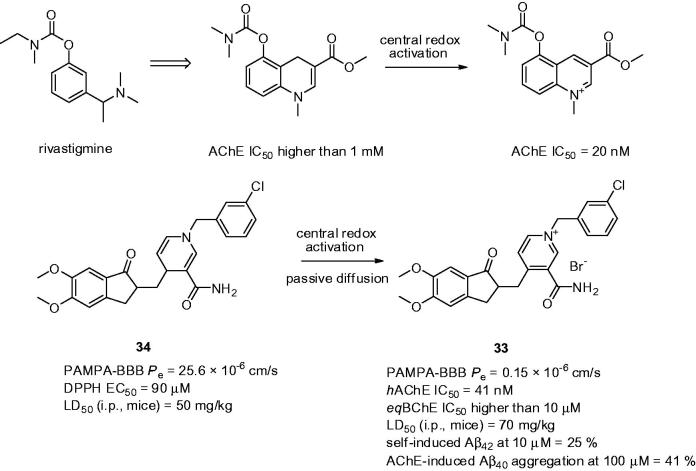
Bio-oxidizable pro-drugs **34** forming charged entities after oxidative activation **33**. The proof-of-concept was firstly validated on rivastigmine-like analogue – the upper part of the Figure.

Bio-oxidizable prodrugs represents interesting concept to relieve the potential side effects. The first-generation of bio-oxidizable AChEI masking the positive charge in rivastigmine was developed intending to transport the pro-drug to the brain avoiding the compounds peripheral side effects. Indeed, the proof-of-concept further revealed the central redox-activation presumably mediated via NADH dehydrogenase resulting into exclusive central cholinergic activation (AChE_peripheral_ IC_50_ > 1 mM; AChE_central_ IC_50_ = 20 nM; Figure 7)^121^. These results encouraged researchers to apply this approach on donepezil template^122^. Accordingly, piperidine moiety was replaced by a 1,4-dihydropyridine ring containing EWG at C-3 position that constitutes a key element/stabilizer in the design of the pro-drug. Importantly, redox-activation step mediated by oxidoreductases does not only convert the pro-drug to pyridinium drug but also entails a “locked-in” effect in the brain. By that it prevents peripherals cholinergic side effects while enabling prolonged duration of AChEI in brain tissue. The majority of the pyridinium salts exhibited potent, *h*AChE nanomolar scale inhibition potency in the same range as donepezil and low inhibitory activity against *eq*BChE. Compound **33** stands out the most interesting from the subset (**33**: *h*AChE IC_50_ = 41 nM; *eq*BChE IC_50_ > 10 µM; Figure 7) showing AChE dual-binding site character. Multipotent profile of **33** was corroborated by inhibition of AChE-induced Aβ_40_ aggregation (41% at 100 µM) and inhibition of self-induced Aβ_42_ aggregation (25% at 10 µM). High permeability value (*P*_e_) of pro-drug **34** over **33** underlined the lipophilic character of **34** to be eligible to permeate through BBB (*P*_e_ for **34 **=** **25.6 × 10^−6 ^cm/s; *P*_e_ for **33 **=** **0.15 × 10^−6 ^cm/s; high BBB permeation is predicted when *P*_e_ value >4.0 × 10^−6 ^cm s^−1^, low BBB permeation equals *P*_e_ < 2.0 × 10^−6 ^cm s^−1^ and uncertain BBB permeation lies in between *P*_e_ value ranging from 2.0 × 10^−6 ^cm s^−1^ to 4.0 × 10^−6 ^cm s^−1^)^123^ ^,^ ^124^. The incubation of **34** in 10% fresh mice brain homogenate led to oxidation product **33** in 35% yield after 180 min. Moreover, **34** exhibited a noticeable radical scavenging activity (DPPH EC_50_ = 90 µM). Under these conditions, **34** is converted into **33** as the main oxidation product which forms robust antioxidant system. Neither **34** nor **33** displayed significant genotoxicity. *In vivo* toxicity evaluation determined LD_50_ values in mice for **34**, **33**, and donepezil as follows – 50, 70, and 22.5 mg/kg, respectively. The repeated administration of **34** at 10 mg/kg did not cause any macroscopic and microscopic tissue alterations thus presuming its relative safety.

### 3.5. Melatonin hybrids

**Figure 8.**
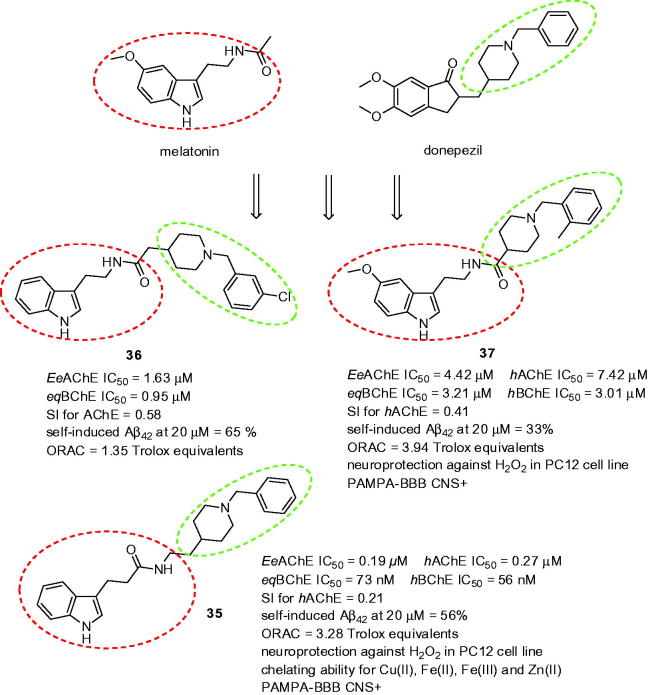
The MTDLs combining melatonin and donepezil templates.

Melatonin (*N*-acetyl-5-methoxytryptamine, Figure 8) is a ubiquitous compound responsible not only for the regulation of circadian rhythm but also for a wide range of other biological activities. This endogenous molecule is able to directly scavenge reactive oxygen and nitrogen species and furthermore, it stimulates the activity of antioxidant enzymes and their expression^125^. Moreover, some studies indicated that melatonin can attenuate tau hyperphosphorylation^126^, reduce the burden of Aβ load^127^, and preclude kainic acid-induced microglial and astroglial responses thus having anti-inflammatory potential^128^. Decreased melatonin in serum and cerebrospinal fluid (CSF) and the loss of melatonin diurnal rhythm are frequently observed in AD patients^129^. Due to the aforementioned benefits, low toxicity and no significant side effects, melatonin is largely used as a scaffold for design of various multifunctional compounds, a number of them being melatonin-donepezil hybrids^130^.

The design of three series of multifunction compounds combining donepezil and melatonin has been reported^131^. Although the design approach was rather simple it produced potent hybrids with a plethora of biological activities. The lead compounds consisted of substituted or unsubstituted *N*-benzyl piperidine and indole fragments joined via a carboxamide linker. All of the synthesised derivatives showed good inhibitory activities against *Ee*AChE and *eq*BChE and their human equivalents, the most potent being **35** (*Ee*AChE IC_50_ = 193 nM; *eq*BChE IC_50_ = 73 nM; *h*AChE IC_50_ = 273 nM; *h*BChE IC_50_ = 56 nM, SI for *h*AChE = 0.21; Figure 8). Kinetic study performed with this ligand indicated a mixed-type inhibition mode. Almost all the compounds exhibited higher activity for BChE than for AChE, thus being more selective BChEIs. Most of the hybrids also displayed moderate to good (33–65%) inhibition potencies for Aβ_42_ self-induced aggregation at 20 µM. Within this context, the most potent inhibitor throughout the subset was **36** (Figure 8). Not surprisingly, good antioxidant properties were achieved with ORAC values ranging between 0.82 and 3.94-fold of Trolox equivalents. The most active with this respect was **37** (3.94-fold of Trolox value; Figure 8) bearing 5-methoxy group at indole ring and 2-methyl group at benzene ring. The capacity to protect the rat pheochromocytoma cell line PC12 cells against oxidative stress-associated death induced by H_2_O_2_ was tested on selected derivatives. All of the compounds exhibited neuroprotective effects at concentrations ranging from 1.25 to 10 µM. Derivative **35** revealed almost the same capacity as Trolox at 10 µM and much better than melatonin. Moreover, **35** demonstrated good biometal-chelating ability towards Cu(II), Fe(II), Fe(III), and Zn(II). Amide bond was designated as potential metal chelating unit in the molecule.

**Figure 9.**
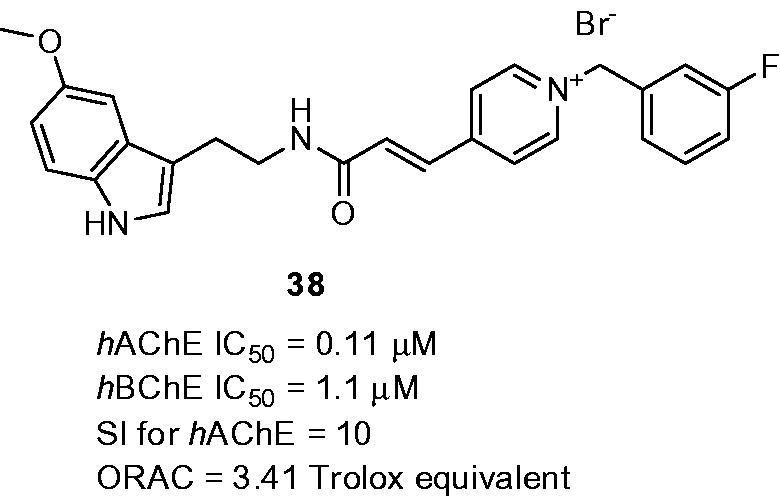
Melatonin-*N*-benzylpyridinium bromide hybrid **38**.

The rational combination of melatonin and *N*-benzylpyridinium bromides as multipotent compounds with enhanced solubility profile was reported^132^. The basic indole core was either substituted with 5-mehoxy group or remained unsubstituted. Interestingly, in both cases, these structural templates preserved remarkable antioxidant properties ranging from 1.14 to 3.66 Trolox equivalents. All the compounds also showed ability to inhibit both human ChEs with preferential inhibition of *h*AChE over *h*BChE. However, the most active analogue in this subset, melatonin-*N*-benzylpyridinium bromide hybrid **38** (*h*AChE IC_50_ = 0.11 µM; *h*BChE IC_50_ = 1.1 nM; SI for AChE = 10; ORAC = 3.41 Trolox equivalent; Figure 9) exerted even 10-fold decreased lower potency to inhibit *h*AChE than donepezil. Further assessments of **38** confirmed its dual binding site character of *h*AChE (mixed-type pattern inhibition, docking studies) and its ability to protect SH-SY5Y cells against oxidative stress induced by H_2_O_2_ *in vitro*. Interestingly, **38** revealed opposite arrangement in the enzyme cavity compared to donepezil which might be responsible for its lower activity.

### 3.6. 8-Hydroxyquinoline derivatives

**Figure 10.**
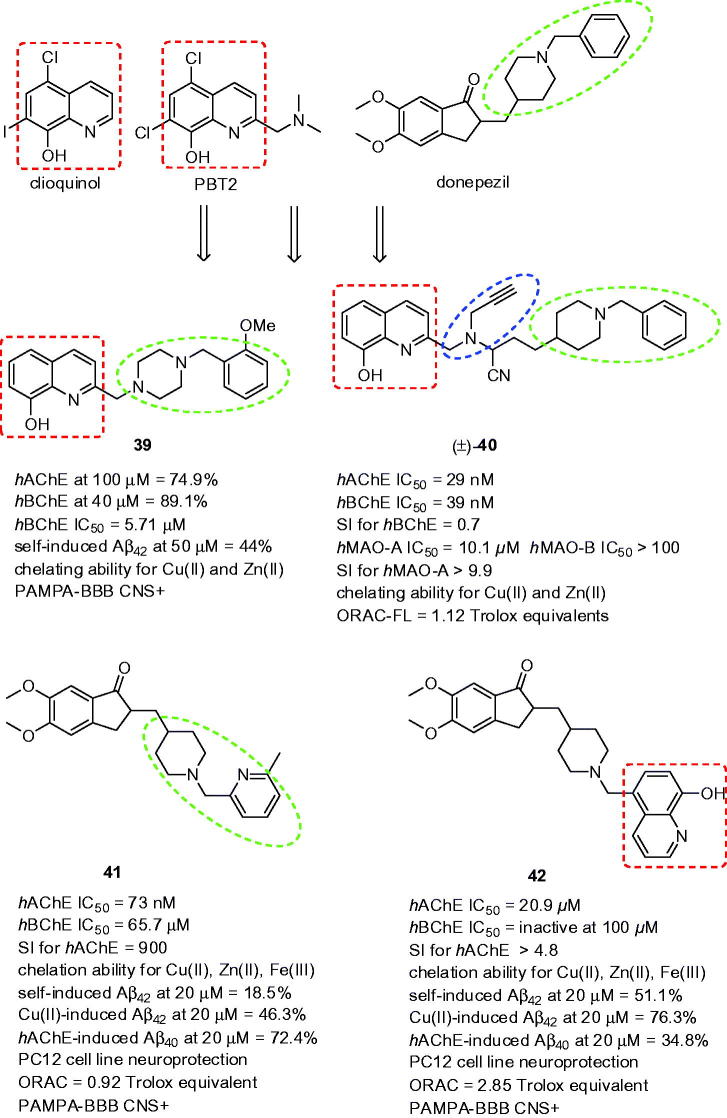
The most potent analogues **39**–**42** related to donepezil-8HQ hybrids.

The combination of donepezil moieties, i.e. *N*-benzylpiperidine or its isoster *N*-benzylpiperazine, with metal chelating core from clioquinol/PBT2 (Figure 10), i.e. 8-hydroxyquinoline (8HQ), resulted in novel multipotent compounds^133^. PBT2 and cliquinol are well established neuroprotective agents with potential therapeutic implication in AD, Parkinson’s disease or Huntigton’s disease^134^ ^,^ ^135^. Authors of the study investigated the effect on cholinesterase activity of EWGs as well as EDGs in different position of benzylic ring of donepezil. Interestingly, initial screening assay revealed that most of these 8-hydroxyquinoline derivatives were *h*BChE selective showing from 49.2% to 89.1% inhibitory potency at 40 µM compounds concentration. Among these hybrids, compounds bearing any substituent at 2-position of benzylic fragment revealed the highest *h*BChE inhibitory activity. Replacing the piperazine nucleus by 4-aminopiperidine one showed drastic effect in term of the *h*AChE affinity enhancement for this enzyme. The authors of the study speculated that this effect might be explained by higher degree of flexibility. The introduction of 8HQ core, mimicking the dimethoxyindanone moiety from donepezil, embedded compounds with neuroprotective properties. In this regard, the authors evaluated Aβ anti-aggregating properties of these compounds reaching moderate to high inhibition potencies (Aβ_42_ = 19.1–65.0% at 50 µM), the highest value was quite close to well-known anti-aggregating compound curcumin (73.7%)^136^. The ability of complexing Cu(II) and Zn(II) was also investigated by UV–Vis difference spectroscopy. The results of this assay for selected compounds showed that these could effectively complex metal ions in physiological conditions, i.e. phosphate buffer, pH = 7.4. Additionally, all the compounds displayed antioxidant ability, some of them even increasing the capability of Trolox in scavenging free radicals. Finally, PAMPA-BBB predicted their potential to reach the therapeutic targets in CNS. In the subset, 8HQ analogue **39** showed balanced multipotent profile (*h*BChE IC_50_ = 5.71 µM; PAMPA-BBB CNS+; Figure 10).

Another series of 8HQ hybrids bearing *N-*benzylpiperidine was enriched with propargylamine fragment to extend the range of biological activities of MAOs-inhibition^137^. This combination led to the development of derivatives with inhibition potency against ChEs and MAOs, metal-chelating capacity and antioxidant properties. Compound with most interesting profile racemic analog (±)-**40** was selected as the drug-lead candidate being *h*AChE/*h*BChE nanomolar inhibitor (*h*AChE IC_50_ = 29 nM, *h*BChE IC_50_ = 39 nM; Figure 10). Moreover, it also showed to be a selective MAO-A inhibitor in comparison to MAO-B (*h*MAO-A IC_50_ = 10.1 µM, *h*MAO-B IC_50_ > 100 µM). The latter was expected based upon the *N-*propargyl moiety attachment^138^. The kinetic studies showed mix-type inhibition for both ChEs and irreversible MAO-A inhibition. Derivative (±)-**40** was also found as metal-chelating agent for Cu(II) and Zn(II). The antioxidant properties of (±)-**40** was carried out by different *in vitro* assays, the most of them showing positive result.

Hybridisation of phenyl ring of *N-*benzylpiperidine moiety by pyridine, quinolone, or 8HQ yielded into novel multipotent donepezil analogs^139^. Mostly, the hybrids exerted prevailing AChE inhibitory selective profile not making the differences, whether small or bulkier substituents were introduced. The most active compound in term of the highest inhibition potency bore 2-fluoro-benzyl moiety (*Ee*AChE IC_50_ = 43 nM, *h*AChE IC_50_ = 32 nM; Figure 10) being more active than template donepezil. Phenyl replacement by pyridylmethyl group in compound **41** (*Ee*AChE IC_50_ = 85 nM, *h*AChE IC_50_ = 73 nM; *Eq*BChE IC_50_ = 20.5 µM; *h*BChE IC_50_ = 65.7 µM; SI for *h*AChE = 900, Figure 10) retained high anti-AChE activity, however, not overwhelming the potency of donepezil. Attaching 8HQ to piperidine moiety had detrimental effect for both AChE and BChE inhibition. Obviously, **41** acted as mixed-type inhibitor of *h*AChE which was also in agreement with the docking protocol. Indanone moiety preferably occupied PAS of *h*AChE via plausible π–π interaction with Trp286 whereas pyridylmethyl-substituted piperidine was anchored to CAS mainly via cation–π interaction to Trp84. The fluorescence emission spectra indicated that **41** is able to complex with Cu(II), Zn(II), and Fe(III). For Cu(II)-**41**, 1:1 stoichiometry was found. When tested at 20 µM, **41** displayed 18.5, 46.3, and 72.4% inhibition of Aβ_42_ self-induced aggregation, Cu(II)-induced Aβ_42_ aggregation and *h*AChE-induced Aβ_40_ aggregation, respectively. Note that the remarkable anti-amyloid profile also achieved the hybrid bearing 8HQ fragment **42** (51.1%, 76.3%, and 34.8% inhibition of Aβ_42_ self-induced aggregation, Cu(II)-induced Aβ_42_ aggregation, and *h*AChE-induced Aβ_40_ aggregation, respectively, Figure 10). The crosstalk between Aβ and **41** and **42** was also corroborated by the data from cell viability and neuroprotection against Aβ_42_-induced and Cu(II)-Aβ_42_-induced toxicity assays indicating that these hybrids possess neuroprotection by improving cell viability in PC12 cell line. Based on the structure aspects, **42** displayed more potent antioxidant scavenging properties than **41** (ORAC-FL = 0.92 and 2.85 Trolox equivalent for **41** and **42**, respectively). Before *in vivo* testing, compounds were tested for their potential permeation through BBB using PAMPA-BBB assay. As expected, **41** and **42** showed satisfied potential for BBB penetration. Mice treated with **41** (10 mg/kg) demonstrated comparable results to that of donepezil administered at 5 mg/kg to reverse scopolamine-induced memory deficit in the step-through passive avoidance test. No alterations were observed in the levels of AST and ALT suggesting **41** as a safe compound.

### 3.7. Donepezil-based derivatives of glutamic acid

**Figure 11.**
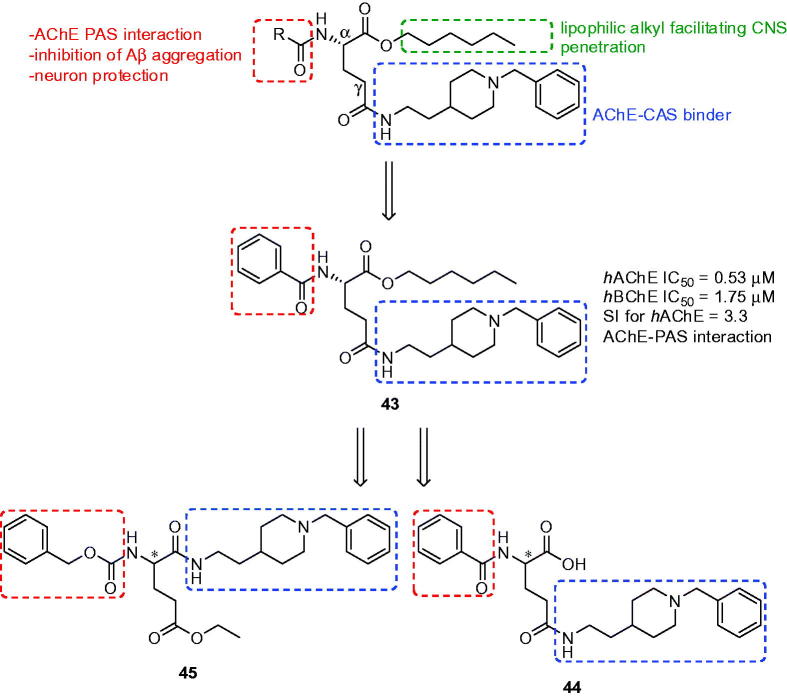
Donepezil-based derivatives with glutamic acid.

An interesting approach to novel multifunctional donepezil-based derivatives for the treatment of AD was applied by using glutamic acid as a suitable biocompatible linker^140^ ^,^ ^141^. This α-amino acid was chosen because of the appropriate distance between α-NH and γ-COOH groups that allows simultaneous interaction for the pharmacophoric groups within the CAS and PAS of AChE. The three fragments were coined with l-glutamic acid in the first family of these derivatives exploiting: (i) *N*-benzylpiperidine moiety from donepezil chosen to inhibit CAS of AChE; (ii) *N*-protecting group able to interact with PAS of AChE along conferring neuroprotection against oxidative stress; and (iii) a lipophilic alkyl ester that would facilitate penetration into the CNS through BBB (Figure 11)^140^. All the prepared compounds showed good inhibition of the *h*AChE with IC_50_ values in the sub-micromolar range (0.10–0.53 µM). They also inhibited *h*BChE with IC_50_ in micromolar scale. The majority of the measured analogues showed significant ability to displace the propidium cation from the PAS thus suggesting its ability to inhibit Aβ aggregation promoted by AChE. The *in vitro* PAMPA-BBB assay showed *P*_e_ values of measured glutamic acid derivatives over the CNS + limit presuming their passive permeation. The authors also studied cell viability and neuroprotective effects of synthetised derivatives against death induced in human neuroblastoma cell line SH-SY5Y by various toxic insults related to oxidative stress triggered by H_2_O_2_ and the mixture of rotenone and oligomycin A (R/O). According to the results all tested compounds protected cells from damage induced by H_2_O_2_ and were able to protect neuroblastoma cells against both exogenous and mitochondrial ROS^140^. Compound **43** (Figure 11) was selected for further pharmacological evaluation. It displayed voltage-gated calcium channel blockade^142^ infarct volume reduction in a photothrombotic stroke-model in mice and neuroprotection against sodium and calcium overload in motor neuron-like NSC-34 cells, as a model of amyotrophic lateral sclerosis^143^ ^,^ ^144^.

Hybrid **43** was chosen as a lead compound for the design of second series of l- and d-Glu *N*-benzylpiperidine derivatives (Figure 11). All new compounds showed good inhibition ability of both human ChE with IC_50_ values in the low- and submicromolar range. The amino acid chirality seemed to have little influence on the *h*AChE inhibition, with the exception of l-**44** (Figure 11) that was two orders more active than d-**44**. All tested compounds displayed preference for *h*AChE with the exception of enantiomers derived from **45** (Figure 11) with *N*-benzylpiperidine fragment in the α-carboxyl group where d-isomer proved 10-fold better *h*BChE inhibition profile over l-**45**. The majority of the compounds displayed neuroprotection behaviour ranging from 13% to 46% using R/O as toxic insult, and from 32% to 52% in OGD/R protocol in human neuroblastoma cell line SH-SY5Y. In both models, l-isomers displayed better protection than their d-counterparts, the best neuroprotection values showed compound l-**44**. Tested compounds were inactive at 10 µM as antioxidants, with the exception of derivatives bearing indole-3-carbonyl moiety that showed radical clearance around 70–73% of the Trolox value. However, the good neuroprotective properties of these compounds towards counteracting free radicals could be presumably associated to the activation of endogenous antioxidant pathways rather than their ability to capture these radicals. Compound l-**45** was, like in the case of l-**43**, chosen for additional pharmacological assays based on its balanced biological profile and high predicted CNS-permeability. Derivative l-**45** displayed voltage-gated calcium channel blockade being 6-fold less potent than l-**43**. On the other hand, in the neuroprotection study in a tissue model of cerebral ischemia, compound l-**45** was more efficient than the reference compound l-**43**.

### 3.8. Lipoic acid hybrids

**Figure 12.**
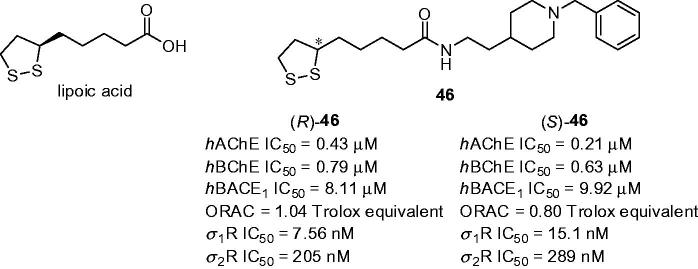
Structures of lipoic acid and the most active lipoic-*N*-benzylpiperidine hybrids (*R*)-**46** and (*S*)-**46**.

Lipoic acid (LA, Figure 12) is a natural antioxidant with ability to scavenge free radicals in both membrane and aqueous domains^145^. These features are mostly attributed to chelating redox-active transition metals, increasing the levels of reduced glutathione and down-regulating inflammatory processes. Besides having direct antioxidant potential, the relevance for using LA in drug design is underlined by the fact that it can also regenerate other biogenic antioxidants^146^. Therefore, LA is considered as privileged scaffold in designing novel biologically active compounds. One such series composes of LA with attached *N*-benzylpiperidine hybrids (Figure 12) to target three AD therapeutic objectives: (i) AChE; (ii) β-secretase-1 (BACE1) and (iii) σ-1 receptor (σ_1_R)^147^. Initial hits derived from LA demonstrated ability to reduce oxidative stress and provide neuroprotection^148^. Novel family displayed moderate inhibition of *h*ChE with IC_50_ values in the micro- to sub-micromolar range^147^. The best inhibition was demonstrated by derivatives **46** with two carbon linker between lipoic acid and *N*-benzylpiperidine moiety. The inhibition activity of racemic mixture (*R*,*S*)-**46** (*h*AChE IC_50_ = 0.39 µM, *h*BChE IC_50_ = 1.23 µM) and enantiomers (*R*)-**46** (*h*AChE IC_50_ = 0.43, *h*BChE IC_50_ = 0.79 µM, Figure 12) and (*S*)-**46** (*h*AChE IC_50_ = 0.21 µM, *h*BChE IC_50_ = 0.63 µM, Figure 12) did not show any noticeable activity differences. Each enantiomer of **46** was found to be good *h*BACE1 inhibitors with IC_50_ values in the low micromolar range, the best being however the racemic mixture with *h*BACE1 IC_50_ =  5.65 µM. According to PAMPA-BBB assay both enantiomers were predicted to reach CNS. The affinities of LA-based analogues **46** for *σ*_1_R and *σ*_2_R were determined in competition experiment with radio-ligands. Tested compounds showed good affinities for *σ*Rs, with *K*_i_s between the low-micromolar and the low-nanomolar scale. The racemic mixture and both enantiomers exhibited preference for the *σ*_1_R subtype with the selectivity ratio against *σ*_2_R > 19. The neurogenic studies on enantiomer (*R*)-**46** to assess its potential ability to promote differentiation of rat brain stem cells into a neuronal phenotype demonstrated that (*R*)-**46** was able not only to promote early neurogenesis but also stimulated neuronal maturation.

### 3.9. Other donepezil hybrids with antioxidant properties

**Figure 13.**
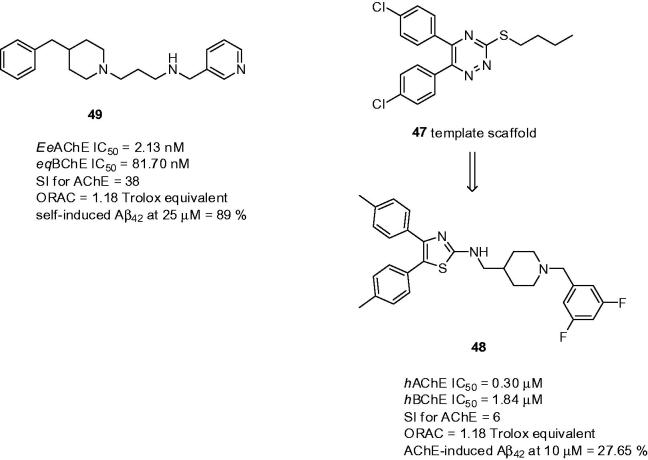
The most active AChEIs **48** and **49** from *N*-benzylpiperidine-diarylthiazole, 5,6-dimethoxy-indanone benzenamides, and *N*′-(4-benzylpiperidin-/piperazin-/benzhydrylpiperazin-1-yl)alkylamine families, respectively.

The design of *N*-benzylpiperidine and diarylthiazole-fused hybrids was based on the previous work where authors have designed and developed vicinally substituted diaryltriazines **47** (Figure 13)^149^. The design of the novel hybrids replaced the morpholino/piperazinoethyl side chain by *N-*benzylpiperidine fragment that was bound to diaryltriazine moiety^150^. It was presumed that diaryltriazine would mimic dimethoxyindanone of donepezil being able to reside in the PAS of AChE. In general, the study disclosed 45 novel compounds combining two basic moieties either via aminomethylene or carboxamide linker. The most promising one was **48** that showed inhibitory activity against both ChEs in sub- and low-micromolar range (**48**: *h*AChE IC_50_ = 0.30 µM, *h*BChE IC_50_ = 1.84 µM, Figure 13), respectively, and exhibited potent inhibition of AChE-induced Aβ_42_ aggregation at 10 µM concentration (27.65%). The enzyme kinetic study of **48** indicated a mixed type of AChE inhibition being in line with the design of these hybrids. The PAMPA assay predicted its ability to cross the BBB by passive diffusion. The effect of **48** on cell viability and neuroprotective potential against oxidative stress were evaluated using the human neuroblastoma SH-SY5Y cell line. Compound **48** imposed negligible cytotoxicity even at 80 µM concentration to the human neuroblastoma SH-SY5Y cells and showed 39.6% neuroprotection (SH-SY5Y cells, tested at 10 µM of **48**; H_2_O_2_ insult). In the DPPH assay **48** was found to exhibit free radical scavenging activity (55 and 70% at 10 µM and 20 µM concentrations, respectively). These *in vitro* results prompted following *in vivo* studies. Derivative **48** showed ROS scavenging and antiapoptotic properties against Aβ_42_-insult in the primary rat hippocampal neuron cultures. Intracerebroventricular injection of Aβ_42_ to rats worsened hippocampal-dependent memory working which was improved when treated with **48**. The treatment with this drug attenuated the antiamnestic effect cause by scopolamine. Moreover, it also reduced the brain levels of malondialdehyde suggesting its antioxidant properties with simultaneous catalase level elevation. **48** also significantly attenuated the levels of Aβ_42_ and *p-*tau and demonstrated significant antiapoptotic potential by lowering the levels of cleaved caspase-3 and cleaved-poly(ADP-ribose) polymerase-1 (PARP) as assessed by Western blot analysis. According to the pharmacokinetic analysis, **48** has good oral absorption and elimination at moderate rate compared to the absorption phase.

*N*′-(4-Benzylpiperidin-/piperazin-/benzhydrylpiperazin-1-yl)alkylamine derivatives were developed to target the multifaceted nature of AD^151^. The 4-benzylamine motif was combined with an appropriate alkyl spacer of varying length with electron rich substituents such as hydroxy, di-, and tri-methoxy groups on the aromatic ring and/or a nitrogen-containing heterocyclic system. These were previously shown to play an important role in inhibiting Aβ aggregation and scavenging of a variety of reactive oxygen species^152^. Regarding ChE inhibition, the SAR studies revealed that 4-benzylpiperidine derivatives bearing a terminal pyridine ring and a vanillyl group on the benzyl ring were the most effective in inhibiting both ChEs, with compound **49** (*Ee*AChE IC_50_ = 2.13 nM; *eq*BChE IC_50_ = 81.7 nM; SI for AChE = 38; Figure 13) being the most potent AChEI in the series. Kinetic analysis with **49** indicated mixed-type inhibition. The ability to inhibit self-induced Aβ_42_ aggregation at 25 µM ranged between 54 and 89%; the most active compound was **49**, displaying anti-amyloid properties at the upper limit. Docking studies performed with **49** on *Ee*AChE revealed significant interactions in the CAS and PAS binding sites, as the pyridine ring was firmly bound to the catalytic site of AChE in the bottom of the gorge, through a favourable π–π interaction with active site residue Trp84. At the cavity entrance, 4-benzylpiperidine was stacked against the indole ring of Trp279 through π–π stacking interactions, which is inconsistent with donepezil superimposition. Generally, all compounds exerted strong antioxidant properties ranging between 0.68–4.45 Trolox values. It was shown that the presence of a vanillyl ring at the terminal end is crucial for providing strong radical scavenging ability. The predicted ADME properties revealed that all compounds fulfil drug-like criteria, exhibit low *in silico* possible toxicity risk, and could be considered as novel nootropic agents based on prediction of activity spectra for substances (PASS) prediction^153^.

## 4. Conclusion

AD constitute the major health problem in the world. The estimated number of patients needing treatment is 40 million worldwide with expectation to nearly double by 2040^3^. Owing to the alarming number of AD patients and population ageing, it is clear that effective cure is urgently required. Currently, marketed drugs provide only symptomatic and short-term relief and their effectiveness do not treat the cause of the dementia^9^.

Compelling evidence imposed AD as multi-factorial disease. Its pathogenesis is composed of many different mechanisms, interaction among them generates a highly complex web of different pathways^154^. Based upon multifactorial aetiology of AD, it is clear that it can be hardly treated by administration of drug targeting solely one pathological condition. For almost three decades, the attention was paid to AChEIs. The cholinergic hypothesis was during this period slightly modified, the paradigm shifted from solely AChEIs towards BChEIs or non-selective ChE inhibitors. This is caused by the fact that cortical levels of BChE show a significant increase in AD progression^155^. Moreover, high BChE levels are associated with the formation of neuritic plaques and neurofibrillary tangles^156^. From this point of view, the marketed drug rivastigmine possesses favourable profile offering dual and sustained AChE/BChE inhibition^157^. On the other hand, when other hypothesis dealing with the onset and progression of AD have been postulated like Aβ and tau hypothesis, it is evident that pharmacodynamics and pharmacokinetic properties of ChEIs are not sufficient to tackle AD. For instance, further studies with galantamine demonstrated its ability to allosterically modulate *α*7 homomeric and *α*4β2 heteromeric nicotinic ACh receptors to alleviate some of the cognitive deficits associated with AD^158^. Intriguing features also favour using donepezil for the treatment of AD. Since its approval, the secondary non-cholinergic role to down-regulate formation of toxic Aβ oligomers and to decrease brain tissue Aβ plaques has been formulated^159^.

Based on data from plethora epidemiological studies several compounds with antioxidant activity have already entered to clinical evaluation to uncover their potential in prevention of cognitive decline and treatment of mild cognitive impairment in AD. Among them, curcumin, LA and other antioxidants not mentioned in this review like vitamin E, resveratrol, ubiquinone (coenzyme Q10), and pramipexole have been clinically investigated. The most of the compounds reached Phase I and II, with the exception of resveratrol. Although available data do not warrant the doubtless use of antioxidants in AD, they are hampered by extremely poor comparability. Moreover, the major profit of radical-free scavengers may lie in their administration in prodromal stage of AD. Thus, the absence of substantial clinical benefit of antioxidants in AD is not disproved to date.

MTDLs have emerged as a promising approach for management of AD^160^ ^,^ ^161^. Several ways how to build these novel and multi-potent ligands exist. From this perspective, framework combination is mostly used either by linking, fusing, or merging two entities in order to implement two or more abilities into single molecule^162^. Several issues are commonly associated with MTDLs designing/development when created by linking or fusing based upon their poor drug-likeness. Being concerned by this fact, we are aware that the true MTDLs will have to possess balanced physico-chemical properties^163–165^. Another challenging task is to obtain MTDLs with balanced activity/affinity profile towards different targets. This can be nicely described as a pair of scales where one activity improvement mostly results into loss/decrease of other. Thus, those who are involved in drug development process like medicinal chemists, pharmacologists, or biochemist have to bear in mind that compounds needs to be optimised by addressing activities in the same concentration ranges. From this point of view, only a very few MTDLs with well-balanced activities/affinities aimed at neurodegenerative disorders are reported today like triazinones (dual BACE-1 and glycogen synthase kinase-3β inhibitors) aminobenzimidazoles (dual BChE and human cannabinoid subtype 2 receptor agonists) or the most advanced drug ladostigil (clinical trial phase 2, AChE/BChE, monoamine oxidase B inhibitors)^138^ ^,^ ^166^ ^,^ ^167^. This is, however, not the case of almost all the ligands reported within this review and thus the true hybrid combining balanced physico-chemical characteristics, antioxidant, and anti-cholinesterase properties is eagerly awaited.

In this review, we have turned our attention to profiling donepezil scaffold into novel chemical entities by conferring the antioxidant properties. The unique features of donepezil chemical scaffold allowed the chemist to mostly retain drug-like characteristics while exacerbate others. Antioxidant profile was mostly achieved by introducing known scaffolds responsible for radical scavenging effect like coumarin, ferulic acid, 8HQ, lipoic acid, and others. It remains speculative whether these agents are able to slow-down the progression of AD acting as symptomatic drugs or they may treat the cause of the disease. In our opinion, the most interesting compounds disclosed herein are bio-oxidizable pro-drug **34** with its activated adduct **33** and “selenpezil” derivatives **21** and **22**, both exerting *in vivo* efficacy. It has to be also noted that oxidative stress closely correlates with inflammation^168^. However, only large clinical studies may provide clear answer as inflammation encompasses dozens of highly interactive molecular mediators and mechanisms, some of them potentially helpful and some of them potentially harmful. Be that as it may, we believe that current knowledge from AD pathogenesis open new route for MTDLs combining ChEIs with antioxidant properties, as the oxidative stress is inherent part of neurodegenerative diseases.
